# In-vivo and in-vitro wound healing and tissue repair effect of *Trametes versicolor* polysaccharide extract

**DOI:** 10.1038/s41598-024-54565-0

**Published:** 2024-02-15

**Authors:** Seyedeh Kiana Teymoorian, Hoda Nouri, Hamid Moghimi

**Affiliations:** Department of Microbiology, School of Biology, College of Science, University of Tehran, Tehran, Iran

**Keywords:** Histopathology, Polysaccharide extract, Scratch assay, *Trametes versicolor*, Wound-healing, Biotechnology, Microbiology

## Abstract

Regarding different medical benefits of fungi, using the medical mushroom extracts as wound-healing agents is gaining popularity. This study, evaluated the wound healing characteristics of *Trametes versicolor*. Anti-oxidant activity addressed by employing the DPPH (2,2-diphenyl-1-picrylhydrazyl) assay resulting 53.7% inhibitory effect. Besides, for anti-microbial ability determination, the MIC (Minimum Inhibitory Concentration) of extract measured which *Escherichia coli* growth was inhibited at 1.1 mg/ml, and *Staphylococcus aureus* did not grow at 4.38 mg/ml of extract. The MTT (3-(4,5-dimethylthiazol-2-yl)-2,5-diphenyltetrazolium bromide) method indicated dose dependence of the extract with 63 ± 3% and 28 ± 3% viability at 1250 μg/ml and 156.25 μg/ml of extract, which higher concentration caused higher cell viability. The outcome of gene expression analysis determined that overall expression of FGF2 (Fibroblast Growth Factor 2), IL-1β (Interleukin-1β), and TGF-β1 (Transforming Growth Factor-β1) was 4 times higher at 48 h than at 24 h in treated cells, suggesting a stimulating effect on cell growth. An in-vivo animal model suggested enhanced wound healing process after treatment with 0.01 g of extract. Furthermore, the number of fibroblasts, epidermal thickness, and collagen fiber was respectively 2, 3, and threefold higher in treated mice when compared to untreated mice. The treated wounds of mice showed 100% and 60% of untreated mice of healing within 14 days. The results of this research show promise for the fungus-based wound healing treatments, which may help with tissue regeneration and the healing of cutaneous wounds.

## Introduction

Every individual faces accident that cause injury and wounds in daily life. It can be created by physical, chemical, thermal, microbial, or immunological events. These wounds will be a burden on people, which is why there have been numerous attempts to develop new therapeutic approaches to reduce the cost and accelerate wound healing^1^. Chemical drugs, such as creams, are the common therapeutic approach to healing wounds. They offer many advantages, including ease of use, anti-microbial and anti-inflammatory properties, and stimulation of cell proliferation^2^. However, there are many disadvantages to these localized creams, such as cell survival after treatment and entering the skin and into each cell^3^. Some antiseptic solutions used in drugs, including hydrogen peroxide, chlorhexidine, and povidone-iodine, have cytotoxicity effects on healthy cells and disrupt the healing process^4^. Concerns exist about the side effects of chemical drugs because they can reach the non-target cells and affect them^2^. It can be concluded that a substitution is needed and natural products with fewer side effects and high effectiveness are proposed.

Because of their anti-oxidant, anti-microbial, and anti-inflammatory qualities, natural products have been the subject of several investigations in recent years that have explored their potential advantages on wound healing^5^. Despite promising wound healing results from medical plants such as Eucalyptus, Aloe vera, curcumin, more study is needed to confirm their effectiveness^6^. Extracts of medical mushrooms are among the most widely used natural products in medicine and are known as mushroom pharmaceutics^7^. This is in terms of their short cultivation time and high concentration of natural components. They possess antibiotic, antitumor, antiviral, immunostimulant and hypolipidemic, anti-elastase, anti-collagenase, anti-hyaluronidase and anti-tyrosinase activities derived from their bioactive compounds, such as polysaccharides, terpenoids, glucans, phenolic compounds, statins, lectins, melanin pigments, chitin and chitosan, which can be found in their cell wall or extracellular or intracellular ways. Particularly important to fungus are polysaccharides, which are also among the best substances for accelerating wound healing^8^. Medical mushrooms can be helpful in the wound healing process by stimulating immune epithelial cells, promoting extracellular matrix formation, triggering cytokines and growth factors, producing reactive oxygen species (ROS), and modulating various inflammatory intermediates^8,9^. Hu et al. in 2023 determined the antioxidant and proliferation effect of *Talaromyces purpureogenus* extract on cells^10^, and another study showed the positive wound healing potential of *Lignosus rhinoceros* extract^11^. Furthermore, a study conducted by Nguyen et al. in 2022, on fruiting bodies of *Cordyceps militaris* revealed 4.1 times faster healing in treated mice during 7 days^12^. Mapoung et al. have indicated that polysaccharide extract of *Auricularia auricular-judae* can enhance cell migration and cell proliferation of HaCat cells; also, it showed accelerated wound closure in wounded mice after 12 days^5^. Polysaccharide extract of *Ganoderma amboinense* has exhibited 144.9% cell survival rate of the HUV-EC-C cell line at 0.2 µg/µl during research of Zhao et al.^13^. Salem et al. have indicated the wound healing properties of ethyl acetate crude extract of *Paecilomyces* sp. (AUMC 15510). They have shown that 5, 10, and 15 mg of extract could enhance wound healing in earthworms after 5 days^14^. In the study that carried out by Yasrebi et al. has been determined that chitosan nanoparticles of *T. versicolor* had the 95% rate of healing in animal models, and fast healing process, which they concluded that it can be used as wound dress^15^. Kaplan et al. revealed that nanoparticle of *T. versicolor* crude had the high cell proliferation and migration ability of L929 cell line at 2.50 μl/ml concentration, by using MTT assay^16^.

This study aims to measure the effect of *T. versicolor* on wound healing in vivo and in vitro, including the anti-microbial, anti-oxidant, cell migration, and gene expression properties of treated cells with *T. versicolor* extract. This research used a novel method to assess the effects of *T. versicolor* polysaccharide extract on wound healing and cell proliferation. Mice were used as the animal model in order to ascertain the pre-clinical outcome for further clinical studies.

## Materials and methods

### Preparation of fungal polysaccharide extract

*T. versicolor* was cultured on Potato Dextrose Agar (PDA) for a period of 14 days. Subsequently, a square segment 1 × 1 cm^2^ of the agar was incubated in Potato Dextrose Broth (PDB) at 150 rpm shaker at 30 °C. Following 14 days, Polysaccharide was extracted by the cold ethanol precipitation method. The broth was mixed with 2 times of 95% (v/v) ethanol volume and incubated at 4 °C overnight. To eliminate residual ethanol, the liquid containing polysaccharide dried out by freeze drying method for 2 days^17^. The powder consisted of impurity such as protein and lipid, besides polysaccharide, which needed to be purified. Trichloroacetic acid methods was employed for protein removal, which it was added to extract at different concentration, followed by centrifuge and drying^18,19^. Chloroform extraction method was used for lipid removal^13^. To qualitatively identify the polysaccharide in final extract, Molisch test were carried out, by using sulfuric acid and α-naphtol. Phenol–sulfuric acid methods conducted to quantify the polysaccharide extract, by establishing the standard curve with glucose dilution^20^. The final brown powder consisted of 40% polysaccharides.

### Assessment of anti-microbial properties of polysaccharide extract

To measure the antimicrobial activity, the Minimum Inhibitory Concentration (MIC) and Minimum Bactericidal Concentration (MBC) methods were used. In the MIC method, 500 μl of Nutrient broth and various concentrations of extract (17.5, 8.75, 4.38, 2.19, 1.1, 0.5, 0.3, 0.1, 0.05, and 0.02 mg/ml) were added. A 25 μl bacterial suspension of *Escherichia coli* ATCC8739 and *Staphylococcus aureus* ATCC 25923 were added to each test tube and incubated at 37 °C for 24 h, then 125 µl of tetrazolium chloride (5 mg/ml) were added to each test tube to evaluate the bacterial growth^21^. The MIC values and higher concentrations were cultured on Nutrient agar and incubated at 37 °C for 24 h in order to perform the MBC process.

### DPPH free radical inhibition assay

DPPH analysis was conducted to measure the antioxidant activity of the extract based on its inhibition of DPPH. Different concentration (2560, 1280, 640, 320, 160, and 80 μl/ml) of the extract was added to 96-well plates. Ascorbic acid was used as a positive control^22^. Following that, 100 μl of the DPPH solution was added to each well in darkness, and after 30 min, the absorbance of the plate was measured by Eliza reader (BioTek Synergy HT) at 517 nm^10,23^. The inhibition percentage was calculated using the following formula:

$$\mathrm{Inhibition \,\%}=({\text{A}}.{\text{C}}-{\text{A}}.{\text{S}})/_{{\text{A}}.{\text{C}}}\times 100$$

A._S_ = Absorbance of sample.

A_.C_ = Absorbance of control.

### Cells and culture preparation

L929 cell line (IBRC C10102) was grown in DMEM (Dulbecco’s Modified Eagle Medium) containing 10% fetal bovine serum and 1% penicillin–streptomycin, which were then incubated at 37 °C with 5% CO_2_ (sab2 Biomedicals MVL-01). After 3 days, the cells were passaged using trypsin Then 3 × 10^3^ cells were seeded in 96 well-plates.

### Cytotoxicity assay

MTT assay was used to evaluate the viability of cells after being treated with extract, and was conducted according to the manufacturer’s instruction (Cib Biotech Co©)^16^. A total of 3 × 10^3^ Cells per well were cultured in a 96-well plate. The extract was filtered by a sterile syringe filter 22 µm (FilterBio®). Different concentrations (1250, 625, 312.5, 156.25 μg/ml) of the fungi extract were added to each well, and incubated for 48 h in 37 °C with 5% of CO_2_. Wells that were not treated with the extract considered as the negative control. After 48 h, MTT reagent was added to each well, and the absorbance of the plate was measured using an Eliza reader (BioTek Synergy HT) at 540 nm^16^.

### L929 cell migration assay

This test was performed to illustrate the migration of cells after treatment with a specific concentration of the extract. A total of 30 × 10^3^ cells per well, without antibiotic, were seeded in a 12-well plate, and the scratch was created in the middle of each well using a sterile tip. The MTT optimal concentration, which was 1250 μl/ml, was added to each well, and incubated at 37 °C with 5% of CO_2_ for 24 h. As a negative control, one well was not treated with the extract. After the cells were incubated for 24 h, they were twice washed with PBS buffer and fixed for 1 h at room temperature with formaldehyde. Following the fixation, a mixture of distilled water and PBS was added to cells for 5 min. The crystal violet that was diluted at ratio of 1:5 with distilled water was added to the wells for 20 min. To wash the residual color, the wells were washed 6 times with tab water. The scratch and migration of the cells were photographed with a microscope (× 100)^24^.

### RNA isolation of samples and quantification real-time PCR

A total of 3 × 10^3^ Cells per well were seeded in a 24-well plate. The cells were treated with 1250 μg/ml of polysaccharide extract, which was the highest and optimal concentration, and incubated at 37 °C with 5% CO_2_. The total RNA was extracted at both 24 and 48 h according to the manufacturer’s instructions^25^. High expression of FGF2^26^, IL-1β^27^, and TGF-β1^28^ (Table 1) would induce cell growth, cell division, and regulation of inflammation, which result in healing, and were used to analyze the effects of the medical mushroom extract on cells using quantitative real-time polymerase chain reaction (qRT-PCR)^29^. To synthesize cDNA from mRNA the M-MuLV reverse transcription enzyme (parstos Iran) was conducted with random hexamers. The test was performed using Real-Time PCR Detection System (Rotor-Gene Q, Biosearch Technologies, Inc, USA), using 2 × SYBER Green Real-Time master mixes (BioFACT, South Korea) regarding the manufacturer’s guidance. The h-HPRT1 used as the internal control gene. Data analysis was carried out using the Comparative Threshold Cycle Method^30^ and the 2^–ΔΔCt^ formula using REST-2009 software^31^, and statistical significance was evaluated using a t-test in the GraphPad Prism software.

**Table 1:** The primers that used to analyze the expression of genes involved in wound healing in L929 cells.

Gene symbols	Primers sequences
TGF-β1	F:TGATACGCCTGAGTGGCTGTCT R:CACAAGAGCAGTGAGCGCTGAA
IL-1β	F:TGGACCTTCCAGGATGAGGACA R:GTTCATCTCGGAGCCTGTAGTG
FGF2	F:AAGCGGCTCTACTGCAAGAACG R:CCTTGATAGACACAACTCCTCTC
h-HPRT1	F:CCTGGCGTCGTGATTAGTG R:TCAGTCCTGTCCATAATTAGTC

### Cutaneous wound animal model

Six male white BALB/c mice, weighing between 18 and 20 g and aged six weeks, were used for the in vivo analysis. All 6 mice were collected randomly, and 4 of them chose with random selection to create the wounds, and separated in two groups, treated ones and positive control. These mice were housed in a roomy (20–25 °C), 30–50% humidity, 12/12 h’ light/dark, air-conditioned animal housing with mouse food, water ad libitum, and wood shavings bedding. The maintenance of animal was in appropriate condition to prevent them from stress. All ethical standards for using animal were followed accordance with ARRIVE guidelines and were approved by Research and Ethics Committee of the College of Science, University of Tehran.

### Creation of excision wound

The procedure of forming the skin wound followed this flow: Four mice were anesthetized using 15 unite insulin syringe with Xylazine® and Ketamine® at a ratio of 3:1 via intraperitoneal injection. Animal’s dorsum was shaved with a razor and commercial depilatory cream. Two circular skin wounds were created by punching the skin with a 5 mm diameter biopsy punch. Aplastic ring was sewn onto the wounds to prevent spontaneous closure^1^. A quantity of 0.01 g of *T. versicolor* extract was applied to both wounds of two mice. The wounds were then covered with sterile gauze and zinc oxide adhesive plaster (SEVA Plast©) to prevent them from being chewed by the mice. As the positive control, three mice remained untreated. For fourteen days, the mice were kept at room temperature at the animal facility, and throughout this time, their wound healing process was continuously observed^5^. For healthy skin analysis, two mice were randomly selected and maintained in the same condition as the wounded mice and positive controls, and considered as negative control. If the condition of the mice’s wound worsened, or the mice died, it was excluded from the study. Due to a covering the wounds by sterile gauze and zinc oxide adhesive plaster, no special treatment was done with the samples receiving the extract. All samples were treated equally and were in the same condition.

### Sample collection from the wound site

After 14 days, the mice were anesthetized and then sacrificed respectively. The Xylazine® and Ketamine® overdose considered as euthanasia method. After making sure that mice are dead, the healed part of the skin was removed, fixed with 10% formalin solution, and stained with hematoxylin and eosin (H&E)^32^ and Masson’s trichrome^33^ for histological and pathological studies. This involved counting of collagen fiber, epidermis, fibroblasts, hair follicles, vessels, and neutrophils. For counting epidermis, fibroblasts, hair follicles, vessels, and neutrophils, sections were stained by H&E, and measured with ImageJ software. The epidermis layer measured by its thickness from dermal–epidermal junction to the surface of the skin in µm scale. Number of fibroblast and neutrophils counted with cell counter plugin in ImageJ. Neutrophils were identified with their plasma granules, which helped accurate counting. To measure the number of hair follicle multi-point tool used in ImageJ, which round shape structures with the presence of cuticle, cortex, and medulla, counted. The area that was corresponding of vessels highlighted by threshold, and all the vessels were count by analyze particles tool. To count and quantify the collagen fiber in cross-sections, the Masson’s trichrome staining employed. Samples stained with Hansen’s iron hematoxylin, then washed with tap water, following staining with Biebrich scarlet-acid fuchsin. After washing with distilled water, samples subjected to phosphomolybdic acid, stained with light green. The final color of collagen fibers was green, nuclei stained blue or black, and cytoplasm seen red. Stained samples visualized using polarized light microscope (40 × and 100 ×), and collagen fiber quantified by image analysis software, ImageJ. In ImageJ software the threshold was manually adjusted to cover the green area, then measured the fibers by setting area and integrated intensity^33^. The stained sections were examined using a digital microscope and photographed at 10 × and 40 × magnifications^5^.

### Statistical analysis

The data were analyzed ImageJ and by GraphPad Prism software and a P-value < 0.05 was considered as a statistically significant level.

### Ethics approval and consent to participate

All animal treatment procedures were performed by the standard ethical guidelines (NIH, publication no. 85–23, revised 1985; European Communities Directive 86/609/EEC) and were approved by Research and Ethics Committee of the College of Science, University of Tehran. All efforts were made to minimize the number of animals used and their suffering.

## Results and discussion

### Antibacterial properties of fungal extract

The MIC of *T. versicolor* polysaccharide extract was 1.1 and 4.38 mg/ml against *E. coli* and *S. aureus*, respectively. The MBC for *E. coli* and *S. aureus* were 2.19 mg/ml and 4.38 mg/ml, respectively (Table 2). The low MIC and MBC values are promising for the prevention of wound infection. The extract was more effective against gram-negative bacteria than gram-positive ones. The anti-microbial activity suggested that this extract had the ability to prevent the growth of both gram positive and gram-negative bacteria. This effect may be carry out by damaging DNA, cell walls, or disrupting enzyme activities with its bioactive products^23^.

**Table 2:** The scavenging percentages of DPPH have determined dose dependence anti-oxidant activity of fungal extract, and MIC results.

Assay	Results
Anti-microbial	Polysaccharide extract MIC (mg/ml)
*E. coli*	1.1
*S. aureus*	4.38

Antioxidant	
Extract concentration (μg/ml)	DPPH inhibition%
2560	53.7
1280	48.3
640	43.8
320	35.9
160	30.4
80	28.2
IC_50_(μg/ml)1300	

The low IC_50_ indicated that polysaccharide extract was highly anti-oxidant. The results of MIC demonstrated that extract was effective to inhibit the growth of *E. coli* and *S. aureus* both bacteria, significantly *E. coli.*

### Anti-oxidant activity of fungal extract

The anti-oxidant activity of extracts was measured and presented in Table 2. The concentration 2560 μg/ml determined 53.7% of DPPH inhibition. Moreover, the IC_50_value of the extracts was $\cong$ 1300 μg/ml, indicating this specific concentration was able to cleavage 50% of DPPH. The findings showed that antioxidant activity was dose-dependent, meaning that a higher extract concentration would also increase its ability to inhibit DPPH. This polysaccharide has immune-stimulate and anti-tumor activities, especially against melanoma and liver cancer, by protecting mitochondria from oxidative stress^22,34, 35^.

### L929 cell proliferation

**Figure 1:**
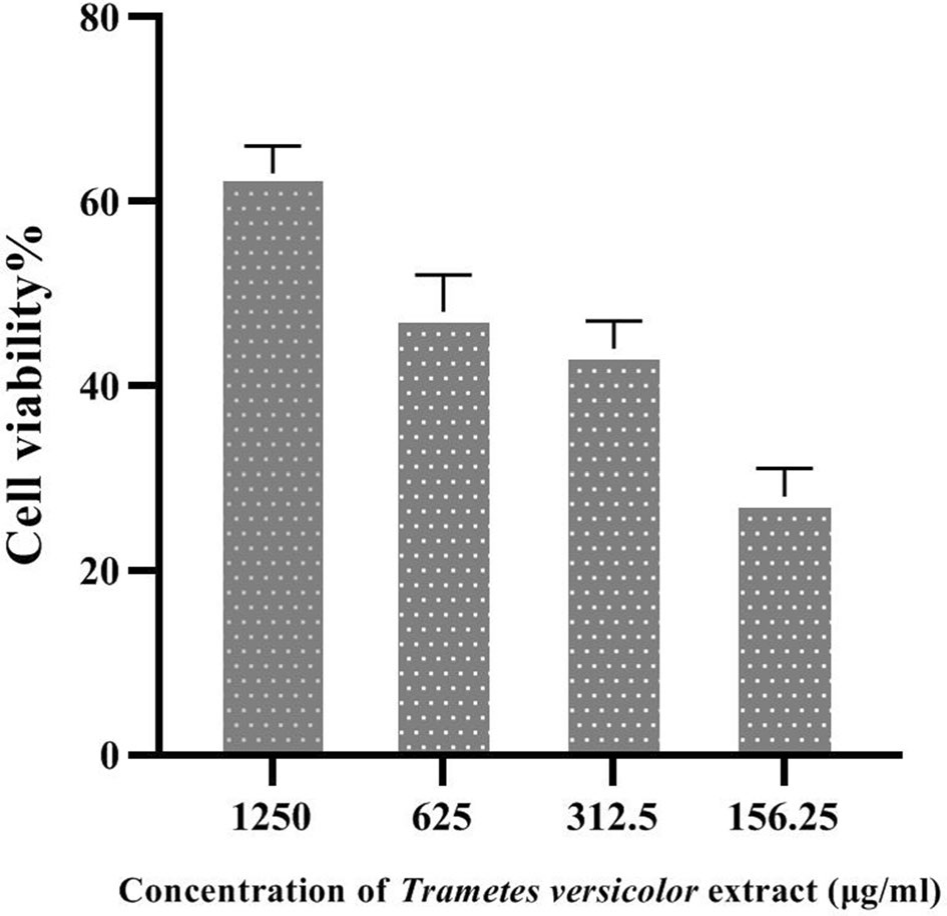
MTT assay. By lowering the concentration of polysaccharide extract cell proliferation and cell growth will be decreased as well. This indicated*Trametes versicolor* extract induces cell cycle and cell proliferation. IC_50_:345.7, R squared: − 1.621, P-value < 0.05.

To demonstrate the impact of the extract on L929 fibroblastic cell viability, the MTT test was conducted. The aim of this test was to indicate the positive effect of fungal polysaccharides on cell proliferation. The results indicated that the extract had induced cell growth in every concentration when compared with the negative control (Fig. 1). Furthermore, the dose-dependent cell growth was obvious, meaning that higher concentration caused more proliferation, meaning that 1250 μg/ml had the most stimulatory effect and cells treated with 156.25 μg/ml showed least cell growths. Not only the fungal extract exhibited no cytotoxic effect on the cells; but also, it stimulated the cell cycle and cell growth, and suggested positive effects on growth. These results would suggest that *T. versicolor* extract has the ability to stimulate cell growth, which might have medicinal uses for tissue regeneration and wound repair. In another study, a polysaccharide extract of 80% (v/v) from *Phliotanameko* exhibited a 5.80-fold higher stimulatory proliferation effect of L929 cells^24^.

### Accelerated migration of L929 cells

**Figure 2:**
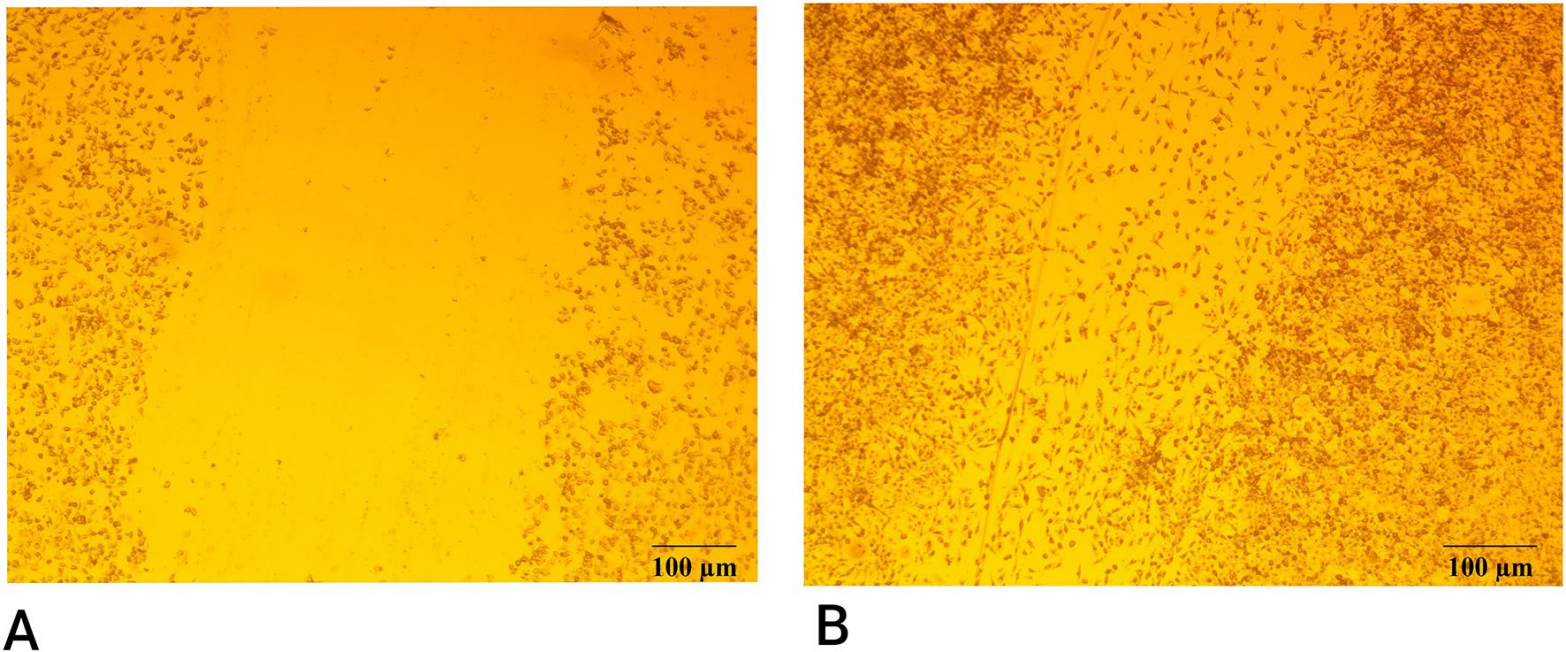
Scratch assay results. (**A**) Negative control of scratch assay. (**B**) Treated cells with extract after 24 h. Treated cells with 1250 µg/ml of polysaccharide extract express high migration and proliferation in comparison with negative control, and 100% closure after 24 h.

After 24 h, microscopic observation of the wells indicated that the scratch of treated cells was filled with cells, covered the created the created scratch, due to a rapid cell proliferation (Fig. 2). This result demonstrated the positive effect of fungal extract on cell proliferation and growth. The cell growth and cell migration of the L929 cell line suggested a potential wound-healing approach. The area of the scratch at T0 (negative control) was 1.5 × 10^7^ µm^2^, which was calculated by wound healing size tool plugin by ImageJ software, and it was completely closed after 24 h, which indicated the 100% relative wound closure after this time period. The specific effect of *T. versicolor* extract depends on the type of extract and the type of cell that is used in a study. For instance, in a study that conducted in 2020, the anticancer effect of *T. vesricolor* lipopolysaccharide extract has been investigated, and it showed anti-migration effect on HUVEC cells and MCF-7 cells^36^.

$$\mathrm{Relative \, wound \, closure\% }=T0-T24/_{T0}\times 100\%$$

T0 = area of the wound at T0.

T24 = area of the wound after 24 h^37^.

### Gene expression analysis after treatment with *T. versicolor* extract

**Figure 3:**
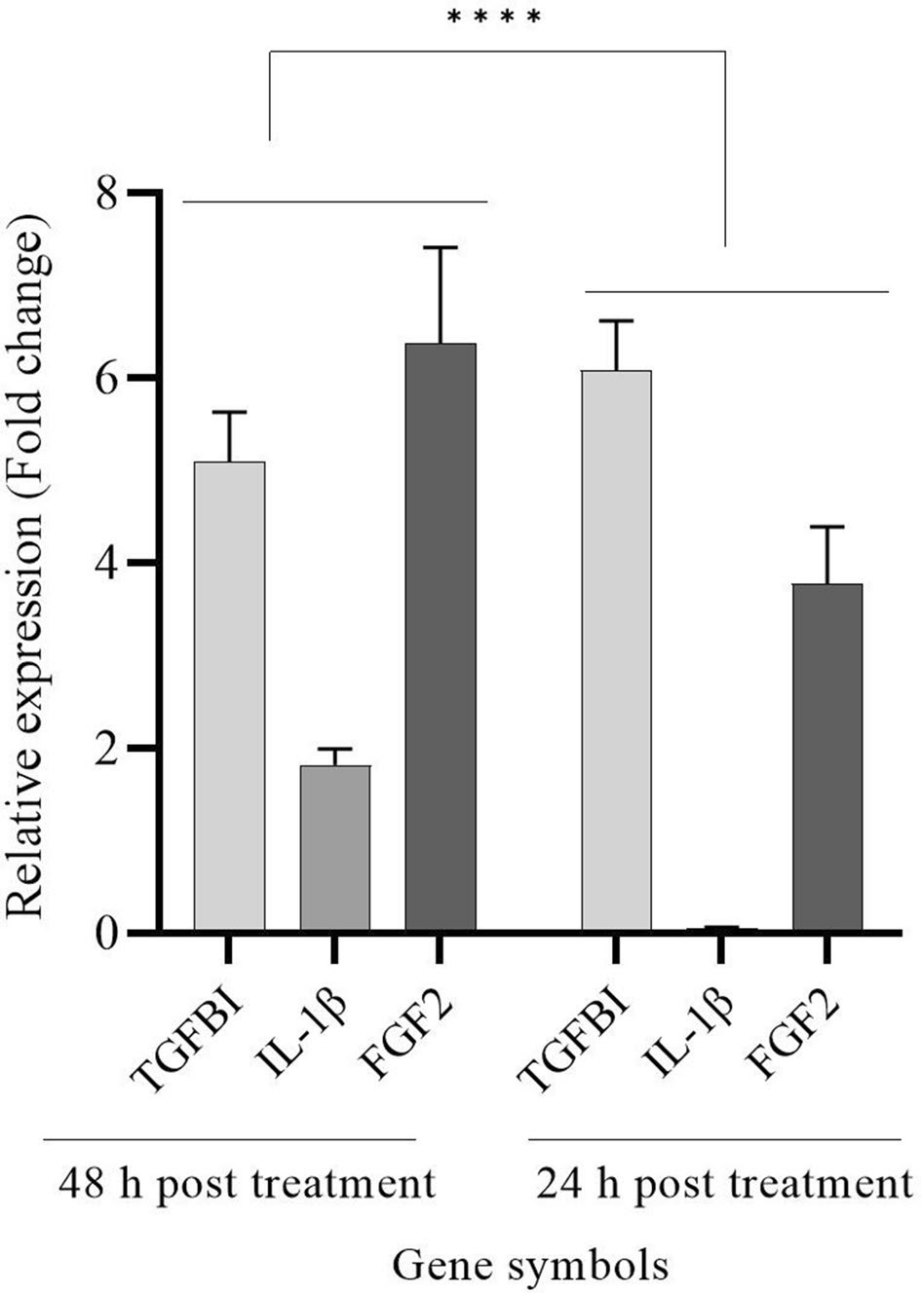
The comparison of expression of TGF-β1, IL-1β, and FGF2 at 24 and 48 h. The expression of TGF-β1 and FGF2 is higher at 24 h, which determined their significant role in early wound healing. At 48 h, IL-1β has expressed more than 24 h. TGF-β1 expression decreased after 48 h. Overall gene expression at 48 h was 4 times 24 h. P-value < 0.05.

The expression of the target genes was evaluated after 24 and 48 h. The results showed in Table 3, TGF-β1expression exhibited 6.08-fold higher at 24 h, FGF2and IL-1β displayed 1.82-fold and 6.38-fold greater expression levels at 48 h, respectively. Notably, FGF2 and IL-β1 showed high expression at 48 h suggesting a time-dependent increase in their expression. However, TGF-β1 expression decreased at 48 h, suggesting that it may play a significant role in the early stages of wound healing and tissue repair. Low expression of IL-β1 at 24 h indicated low inflammation at the first hours of healing process. The high expression of IL-1β at 48 h indicated strong immune response, which could help the prevention of wound infections. TGF-β1 promoted high cell growth and cell division, and faster healing^38^. The genes expressions are illustrated in Fig. 3.

**Table 3:** qRT-PCR results.

Gene symbols	Fold changes	
	24 h	48 h
TGF-β1	6.08	5.09
IL-1β	0.05	1.82
FGF2	3.87	6.38

The fold changes in gene expression at 24 h and 48 h indicated that at early hours of healing the expression of TGF-β1 has been more effective than IL-1β and FGF2expression. On the contrary, at late hours unlike the IL-1β and FGF2expression, the expression of TGF-β1 has been increased.

Polysaccharides have showed the ability to increase the expression of TGF-β1 and to reduce expression in cells, like polysaccharides found in *Caesalpinia ferrea* stem, which had a positive effect on the wound healing process by regulating inflammation^39^. Furthermore, research by Zeng et al. suggested polysaccharide extract from *Cordyceps military* had the ability to activate FGF2/FGFR1c in BaF3 cells, and inducing cell proliferation^40^.

However, Wang et al. showed that underexpression of TGF-β1 helped with redaction of cardiac fibrosis in diabetic mice^41^. An investigation in 2023 showed the anti-melanoma effect of mycelium of *T.versicolor*, which demonstrated that high expression of IL-1β would help the reduction of melanoma^42,43^. Both expression of FGF2 with TGF-β 12.79-fold might increase the cell cycle rate and tissue regeneration^26^. On the contrary, very high expression of these genes was observed in cancer and tumors, so controlled expression is crucial to prevent skin cells from becoming cancerous^43^. Other studies indicated that TGF-β1 and IL-1β overexpression can activate the Smad3 signaling pathway during injuries. This pathway involves repair and fibrosis, so high expression of these genes might cause fibrosis in organs, such as the lungs^44^. These genes may activate several signaling pathways, such as PI3K/PCK, MAPKAPK-2, or TGF-β/SMAD, which induce cell proliferation and wound healing, like 2.5-fold of TGF-β1 expression of rat's cell after 3 days of treated with curcumin nanoparticles^45–48^.

### Appearance examination, histological evaluation, and cell count of treated wound site

**Figure 4:**
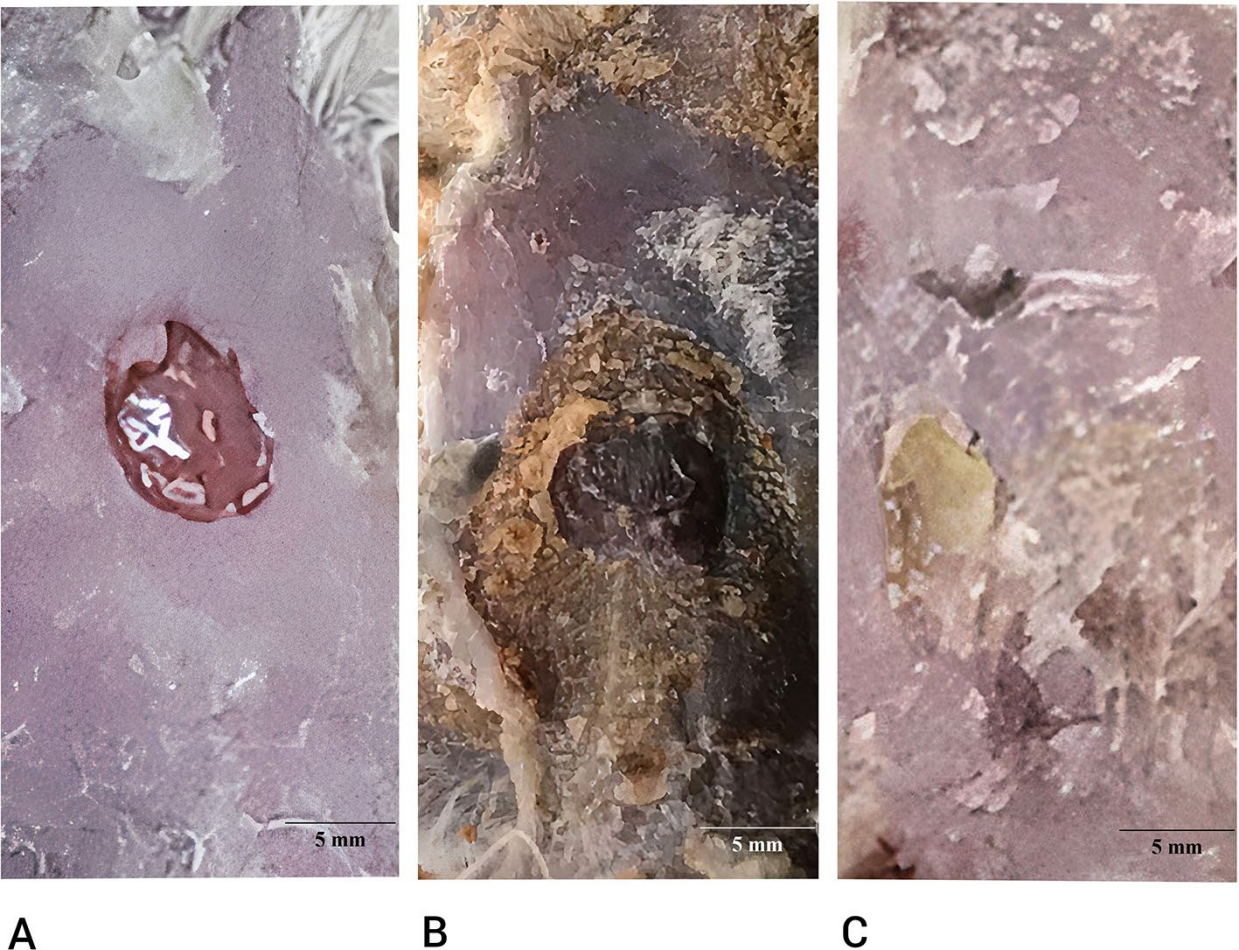
Appearance changes of the wound of control and treated mice in 0 days and 14 days. (**A**) 0 days. (**B**) Negative control after 14 days. (**C**) Treated wound with 0.01 g of polysaccharide extract. Treated wounds showed signs of faster healing and better skin look after 14 days. This determined *Trametes versicolor* polysaccharide extract may have potential wound healing ability.

Animal models used for evaluating the appearance of the created wounds after treatment with the fungal extract. Mice did not show any different in weight, after this period. After 14 days, compared to the positive control groups, the appearance of wounds treated with polysaccharide extract showed a significant improvement and increased healing process, as a result of faster healing (Fig. 4). These indicated the potential tissue regeneration features of this extract. Treated wounds showed no sign of inflammation or apoptosis because of their inflammation regulatory properties after these 14 days, suggesting no cytotoxicity for the cells and the epidermal tissue. As evidence of wound healing, along observation, quantitative analysis showed high percentage of healing. The treated wounds were completely closed, which indicated 100% of healing, and hair growth has been seen. The healing percentage in positive control was 60%, that demonstrated the diameter of 2 mm of wound, which was indicator that the extract accelerated healing process, and no hair growth was seen, after 14 days. Fungal extract demonstrated the ability to increase the epithelial cells and accelerate cell growth in the skin tissue, resulting in faster healing process in treated mice. Untreated wounds showed slight inflammation after 14 days^49^.

$$\mathrm{Healing \, percentage\% }= (1-Wound \, diameter \, at 14th \, day/Initial \, wound \, diameter)\times 100$$

**Figure 5:**
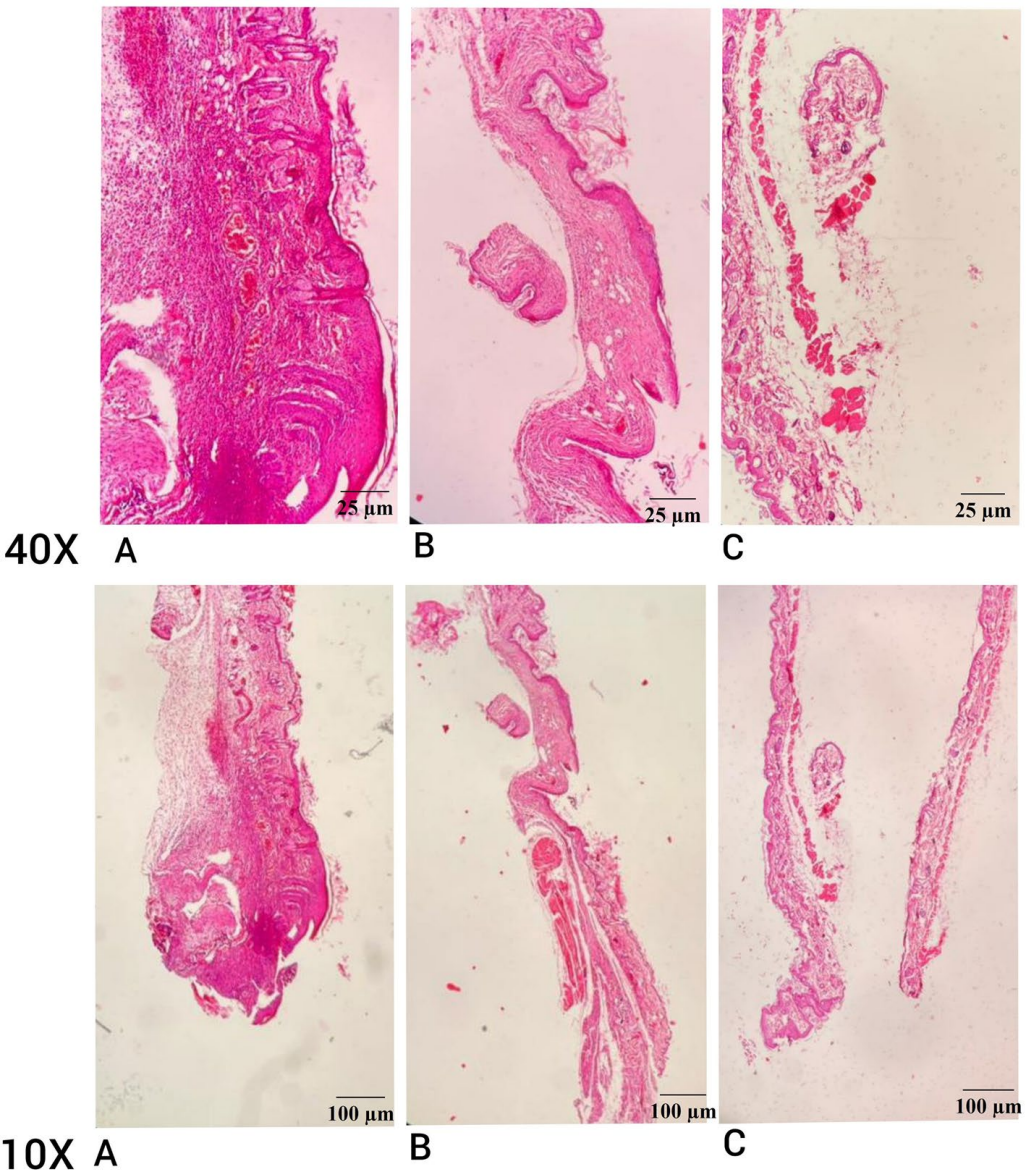
Mice skin cross-segments. (**A**) Untreated punched skin after 14 days. (**B**) Treated punched skin as negative control after 14 days. (**C**) Healthy control skin. Samples are captured at 10 × and 40 × magnifications.

**Figure 6:**
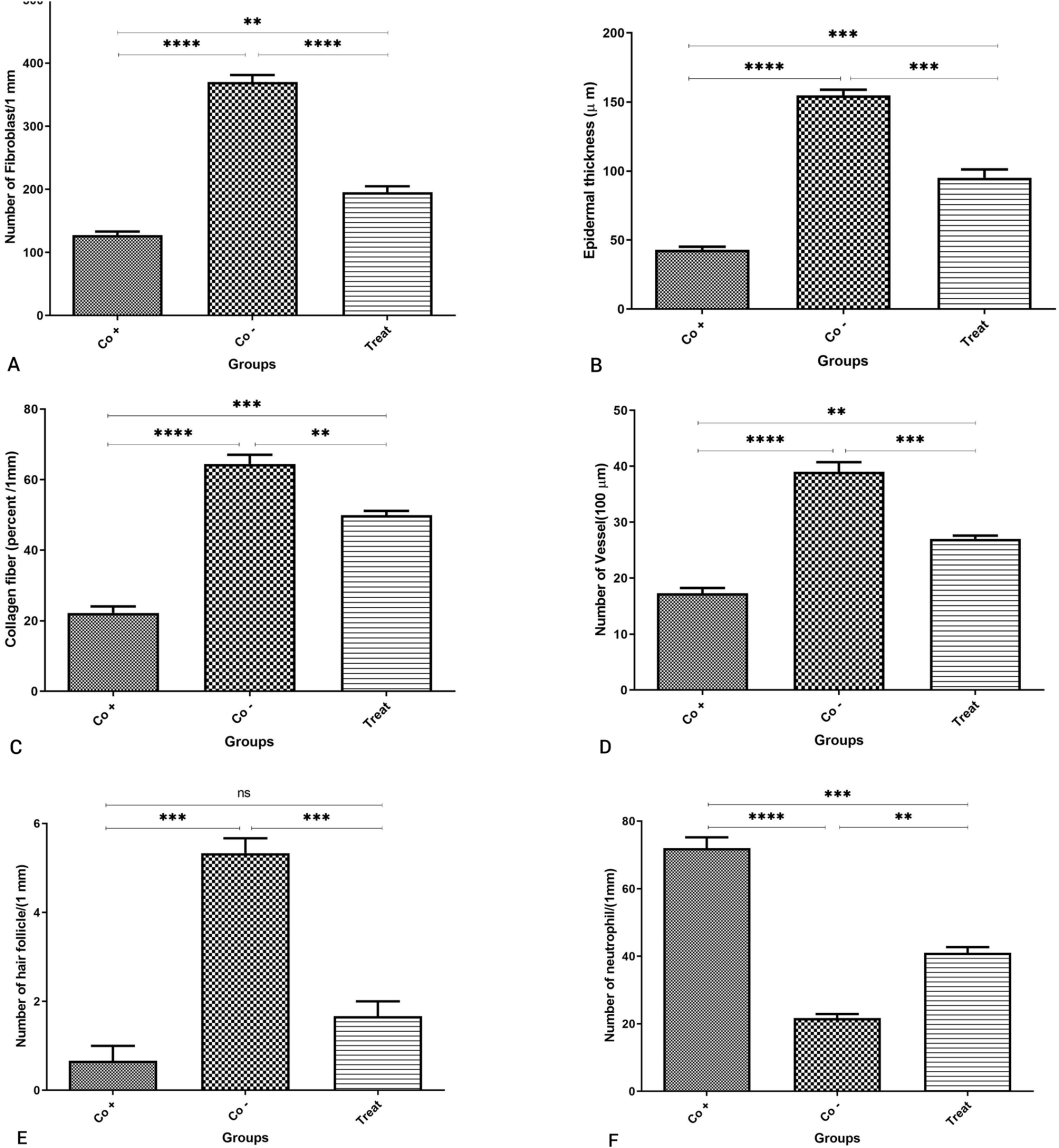
Histopathology results. Fibroblasts (**A**), epidermal thickness (**B**), collagen fiber (**C**), and vessels (**D**) in treated samples observed 2-flold, threefold, threefold, and twofold more than positive control ones after 14 days, respectively. All 4 parameters have been higher in healthy skin. Neutrophil (**F**) was observed higher in positive control than in treated samples, indicating increased inflammation in untreated mice, and hair follicles (**E**) did not show significant differences in treated mice than positive control after 14 days.

Histopathology assessments were conducted to evaluate the efficiency of the polysaccharide extract on healing. Figure 5 shows cross-segments of samples stained with H&E after 14 days. Reduced neutrophils and vessels were anticipated, but a high concentration of fibroblast, thicker epidermal layer, and collagen were the intended results. Collagen indicated the remodeling stage and wound closure, vessels indicated angiogenesis, and the number of neutrophils was a useful signal for assessing the inflammatory phase^1^. Healthy mice were considered the negative control group and untreated groups as a positive control, treated mice sample results were evaluated according to that. Histopathology results, highlighted in Fig. 6, indicated that mice treated with polysaccharide extract had about twofold increase in fibroblast that was responsible for rapid healing rate, with 191 ± 4 fibroblast of treated mice and 122 ± 2 fibroblast of positive mice, which treated wounds had accelerated closure in compare with positive control, a threefold increase in epidermal layer thickness which the thickness of wounds subjected to extract was 93 ± 6 µm, but this thickness for positive wounds was 46 ± 2 µm, that had result in high quality healing process in the mice hat received the extract, and 3-times higher collagen fiber, treated ones with 49 ± 1 fibers and 22 ± 2 fibers of untreated wounds, which was responsible for better appearance of treated mice than untreated ones, and a 2-folds increase in vessels compared to positive control mice, while the number of vessel in treated mice was 27 ± 1, the positive control had 18 ± 2 vessels. Conversely, observations demonstrated slight difference in hair follicles, that treated wounds (1.5 ± 0.5) had more hair growth than untreated ones (0.5 ± 0.5), and a threefold increase in neutrophils in the untreated samples. Treated wounds had 39 ± 3 neutrophils, but number of neutrophils of positive control was 72 ± 6. This significant low level of neutrophils in treated mice in compare with positive control was an indicator for low inflammation level in samples in contact with the extract. As concluded in Mapoung et al.^5^ study, it can be inferred that polysaccharide extract had a positive effect on wound healing and tissue regeneration after treated with extract for 14 days. Higher cell division and collagen formation in the skin would aid a quicker healing process, less inflammation, and a better appearance of skin, and high rate of skin regeneration would be helpful in a short time of treatment and a low dosage of medicine. Additionally, the used extract powder's low weight of 0.01 g indicated good function at a low fungal extract concentration. We infer that a minimal weight of polysaccharide extract may accomplish the intended healing process.

## Conclusion

The promotion and the acceleration of wound healing are significant factors to maintain good health. To achieve this goal natural products such as fungal extract has shown promising wound healing and tissue regeneration properties. The polysaccharide extract from *T. versicolor* has shown encouraging results in this investigation and may have use in the future as a medication for tissue regeneration and wound healing. In conclusion, the *T. versicolor* extract has the potential to increase cell growth and facilitate wound healing. Its ability to stimulate fibroblastic cell proliferation, and the cell cycle, and regulate inflammation through the increased expression of IL-1β, TGF-β1, and FGF2, with its positive impact on healing and tissue regeneration in animal findings, provide promising results for further research and clinical trials.

## Data Availability

All data are included in the manuscript and additional information, and further queries about sharing data can be directed to the corresponding author.
